# Alterations of the cerebral microstructure in patients with noise‐induced hearing loss: A diffusion tensor imaging study

**DOI:** 10.1002/brb3.3479

**Published:** 2024-04-22

**Authors:** Ranran Huang, Aijie Wang, Yafei Zhang, Guochao Li, Yi Lin, Xinru Ba, Xianghua Bao, Yunxin Li, Guowei Zhang

**Affiliations:** ^1^ Department of Radiology Yantaishan Hospital Yantai P. R. China; ^2^ Department of Occupational Yantaishan Hospital Yantai P. R. China

**Keywords:** diffusion tensor imaging (DTI), magnetic resonance imaging, noise exposure time, occupational noise‐induced hearing loss (occupational NIHL), tract‐based spatial statistic (TBSS), white matter

## Abstract

**Objective:**

To explore the changes in the cerebral microstructure of patients with noise‐induced hearing loss (NIHL) using diffusion tensor imaging (DTI).

**Method:**

Overall, 122 patients with NIHL (mild [MP, *n* = 79], relatively severe patients [including moderate and severe; RSP, *n* = 32], and undetermined [lost to follow‐up, *n* = 11]) and 84 healthy controls (HCs) were enrolled. All clinical data, including age, education level, hearing threshold, occupation type, noise exposure time, and some scale scores (including the Mini‐Mental State Examination [MMSE], tinnitus handicap inventory [THI], and Hamilton Anxiety Scale [HAMA]), were collected and analyzed. All participants underwent T1WI3DFSPGR and DTI, and tract‐based spatial statistics and region of interest (ROI) analysis were used for assessment.

**Results:**

The final sample included 71 MP, 28 RSP, and 75 HCs. The HAMA scores of the three groups were significantly different (*p* < .05). The noise exposure times, hearing thresholds, and HAMA scores of the MP and RSP were significantly different (*p* < .05). The noise exposure time was positively correlated with the hearing threshold and negatively correlated with the HAMA scores (*p* < .05), whereas the THI scores were positively correlated with the hearing threshold (*p* < .05). DTI analysis showed that all DTI parameters (fractional anisotropy [FA], axial diffusivity [AD], mean diffusivity [MD], and radial diffusivity [RD]) were significantly different in the left inferior longitudinal fasciculus (ILF) and left inferior fronto‐occipital fasciculus (IFOF) for the three groups (*p* < .05). In addition, the FA values were significantly lower in the bilateral corticospinal tract (CST), right fronto‐pontine tract (FPT), right forceps major, left superior longitudinal fasciculus (temporal part) (SLF), and left cingulum (hippocampus) (C‐H) of the MP and RSP than in those of the HCs (*p* < .05); the AD values showed diverse changes in the bilateral CST, left IFOF, right anterior thalamic radiation, right external capsule (EC), right SLF, and right superior cerebellar peduncle (SCP) of the MP and RSP relative to those of the HC (*p* < .05). However, there were no significant differences among the bilateral auditory cortex ROIs of the three groups (*p* > .05). There was a significant negative correlation between the FA and HAMA scores for the left IFOF/ILF, right FPT, left SLF, and left C‐H for the three groups (*p* < .05). There was a significant positive correlation between the AD and HAMA scores for the left IFOF/ILF and right EC of the three groups (*p* < .05). There were significantly positive correlations between the RD/MD and HAMA scores in the left IFOF/ILF of the three groups (*p* < .05). There was a significant negative correlation between the AD in the right SCP and noise exposure time of the MP and RSP groups (*p* < .05). The AD, MD, and RD in the left ROI were significantly positively correlated with hearing threshold in the MP and RSP groups (*p* < .05), whereas FA in the right ROI was significantly positively correlated with the HAMA scores for the three groups (*p* < .05).

**Conclusion:**

The changes in the white matter (WM) microstructure may be related to hearing loss caused by noise exposure, and the WM structural abnormalities in patients with NIHL were mainly located in the syndesmotic fibers of the temporooccipital region, which affected the auditory and language pathways. This confirmed that the auditory pathways have abnormal structural connectivity in patients with NIHL.

## Graphical abstract

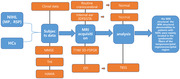

## 1 INTRODUCTION

Sensorineural hearing loss (SNHL) is defined as damage to the sensory hair cells or spiral ganglion neurons with several causes, including neurodegenerative diseases, noise, and ototoxic drugs (Cox et al., 2014; Crowson et al., 2017). Occupational noise‐induced hearing loss (NIHL) is a type of SNHL caused by long‐term exposure to unprotected noise in environments exceeding the limits of national standards (Lie et al., 2016; Loftis, 2007). NIHL is characterized by progressive high‐frequency and bilateral symmetric SNHL that is mainly related to the degeneration and necrosis of hair cells in the inner ear of the cochlea after noise stimulation. Some scholars (Feng et al., 2022; Guo et al., 2021) have reported that NIHL can lead to increased social stress, sadness, decreased confidence, decreased self‐identity, and mental health abnormalities, such as anxiety and depression, among others. NIHL is the second most severe occupational hazard globally (Feng et al., 2022; Yang et al., 2015). It especially affects individuals in developing countries.

Care for NIHL predominantly focuses on clinical behavioral research (blood pressure, endocrine system, cardiovascular system, and even depression and insomnia). The usefulness of neuroimaging studies of brain structural properties and neuroplastic function pathologies in NIHL has been debated upon. Trabanco et al. (2022), in a comparative occupational multifactor study, suggested that noise exposure damages both the peripheral and central auditory systems. Our previous study (Huang et al., 2020) showed that gray matter (GM) volume was significantly different and the auditory cortex underwent functional reorganization in patients with NIHL. Some studies (Hribar et al., 2014; Park et al., 2018) have also reported white matter (WM) macrostructural and microstructural differences in patients with SNHL, including in some auditory regions. Diffusion tensor imaging (DTI) is used to assess tissue integrity. It is mainly used to quantify WM tract water molecule displacement because it reveals more in‐depth changes in the WM microstructure and can deepen our understanding of the subclinical anatomical changes that occur in the auditory pathways of patients with hearing loss (Tarabichi et al., 2018). Some previous DTI studies (Jiang et al., 2019; Karns et al., 2017; Wang et al., 2019) have reported lower fractional anisotropy (FA) in the auditory pathways of patients with common SNHL. These findings suggest that hearing deprivation in patients with SNHL results in axonal or myelin loss. However, knowledge about the microstructural alterations of cerebral WM, specifically in patients with NIHL, is limited.

To determine whether patients with NIHL have the aforementioned discrepancies and address any related controversies, the present study aimed to explore the hypothesis that patients with NIHL have different WM microstructural properties.

## 2 MATERIALS AND METHODS

### 2.1 Participants

Following the Ministry of Health of the People's Republic of China (2014), patients (*n* = 122) who were diagnosed with NIHL by occupational doctors within 2014–2020 were included. The diagnosis and classification criteria were used to divide the patients into the mild patients (MP, *n* = 79) and relatively severe patients (RSP, *n* = 28) (including moderate and severe cases); the severities of 11 patients were undetermined because they were lost to follow‐up. Healthy controls (HCs) (*n* = 84) were also enrolled. The clinical data of all the participants, including age, education level, hearing threshold, occupation type, noise exposure time, Mini‐Mental State Examination (MMSE) score, Tinnitus Handicap Inventory (THI) score, and Hamilton Anxiety Scale (HAMA) score, were collected and analyzed. The MMSE scores were used to assess the cognitive status of all participants (maximum, 30; normal, 27–30; cognitive dysfunction, <27). The THI score was obtained by adding the scores of all 25 items; higher scores were associated with greater tinnitus disability and worse subjective feelings of patients. The HAMA score is based on 14 items, such as anxiety, tension, fear, and insomnia; higher scores indicate more severe anxiety. The following inclusion criteria (Huang et al., 2020) were used: adult male (35–60 years); Han Chinese; primary to university level of education; MMSE scores ≥27 (normal cognitive state); normal mental state with no neuropsychiatric diseases; no systemic diseases or other factors that may affect brain structure or function; and not taking sedatives or central nervous system depressants. Written informed consent was obtained from all participants after a full explanation of the procedures involved. This study protocol was approved by the Yantaishan Hospital Ethics Committee. Informed consent (include appropriate statements) (2023014).

Ministry of Health of the People's Republic of China (2014) is a national diagnostic standard promulgated by the National Occupational Health Standards of the People's Republic of China. Sensorineural deafness was diagnosed based on an occupational noise operation history of more than 3 years, progressive hearing loss, tinnitus, and other symptoms, and pure tone audiometry results combined with occupational health monitoring data and on‐site occupational health investigation, comprehensive analysis, and exclusion of other causes of hearing damage. The average hearing thresholds for the binaural high frequency (3000, 4000, and 6000 Hz) (>40 dB), better whisper frequency (500, 1000, and 2000 Hz), and high frequency (4000 Hz) were used for diagnosis and diagnostic classification: mild—26–40 dB; moderate—41–55 dB; severe: ≥56 dB. For this standard, “noise work” refers to work in an environment with noise intensity exceeding the “occupational exposure limit for hazardous factors in the workplace,” which is an 8‐h‐equivalent sound level (A weight) of >85 dB.

### 2.2 MRI acquisition

Routine craniocerebral and internal ear MR examinations were performed using a GE Discovery MR 750 3.0 T scanner (General Electric Company). Brain structure was examined from sagittal scans using a T1WI 3D‐FSPGR sequence with the following parameters: TR = 6.9 ms; TE = 3.4 ms; 1‐mm section thickness; no gap and 172 slices covering the entire brain; FOV = 25.6 × 25.6 cm^2^; matrix = 256 × 256; NEX = 1; and flip angle = 12°. The DTI (with echo planar imaging) sequence used the following parameters: *b* = 0, 1000 s/mm^2^, TR = 5500 ms; TE = minimum; 3‐mm section thickness; no gap; FOV = 24 × 24 cm^2^; matrix = 128 × 128; NEX = 1, flip angle = 90°; 50 directions of diffusion gradients; and 47 slices covering the whole brain.

### 2.3 Data processing

#### 2.3.1 Whole brain tract‐based spatial statistics (TBSS)

The DTI data were analyzed using the FMRIB Software Library tools (www.fmrib.ox.ac.uk/fsl/) (Smith et al., 2004; Wang et al., 2019). First, all DTI images were corrected for head motion (head motions exceeding 2.0 mm and head rotation angles exceeding 2° were deleted) (Luan et al., 2019) using eddy current correction and linear registration for data preprocessing. FA maps were generated by brain extraction using the Brain Extraction Tool and entered into the FMRIB Diffusion Toolbox (Wang et al., 2019). According to a previous method (Smith et al., 2006; Wang et al., 2019), the FA data of all the participants were registered or co‐registered to the target image using nonlinear registration in the FMRIB nonlinear registration tool and affine‐aligned into the Montreal Neurological Institute 152 standard space. A mean FA image was created and thinned to generate a mean FA skeleton of the WM tracts (Wang et al., 2019). The FA threshold of >0.2 defined the border of the central major fiber bundles. Voxel‐wise FA analysis identified differences among the three groups using a general linear model framework using one‐way analysis of variance (ANOVA) and a nonparametric test with 5000 permutations. Age and education level were used as covariates in the FMRIB Software Library. *p*‐Values of <.05 denoted statistical significance and were corrected using the threshold‐free cluster enhancement method. The individual FA, mean diffusivity (MD), axial diffusivity (AD), and radial diffusivity (RD) maps were generated and analyzed as previously described (Wheeler‐Kingshott & Cercignani, 2009).

#### 2.3.2 Region of interest analysis

**FIGURE 1:**
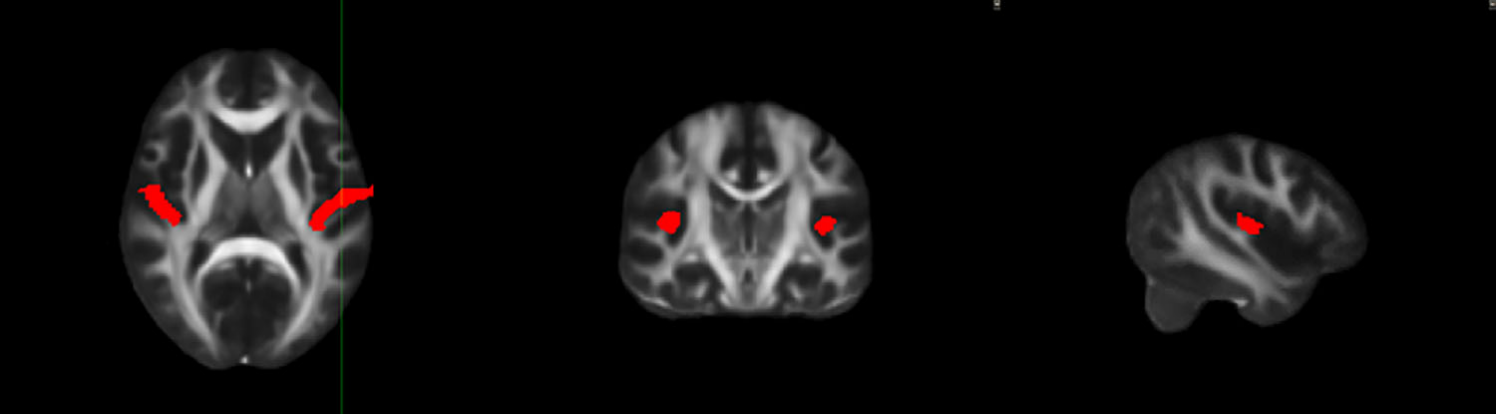
The white matter (WM) tracts of interest Heschl's gyrus selected for region of interest (ROI) analysis. AAL, anatomical automatic labeling.

The region of interest (ROI) analysis focused on the bilateral auditory cortex (Heschl's gyrus [HG]), as shown in Figure 1 (Jiang et al., 2019). The mean DTI values were extracted from the HG. Differences in the FA, MD, AD, and RD values among the three groups were determined using a one‐way ANOVA (*p* < .05).

### 2.4 Statistical tests

The differences in the ages, education levels, MMSE scores, and HAMA scores of the three groups were analyzed using ANOVA. The hearing threshold, noise exposure time, and THI and HAMA scores for the MP and RSP were analyzed using two‐tailed independent sample *t*‐tests (*p* < .05). Bivariate Pearson correlation analysis was performed for clinical capital data (*p* < .05). Differences in the DTI parameter values of the three groups were analyzed using ANOVA (*p* < .05). The Bonferroni test was used for multiple comparisons of the groups (*p* < .0167). Bivariate Pearson correlation was used to analyze the correlation between DTI parameters, hearing threshold, noise exposure time, and HAMA score (*p* < .05). All the data were normally or nearly normally distributed (SPSS Statistics 19.0 software).

## 3 RESULTS

### 3.1 General information

**FIGURE 2:**
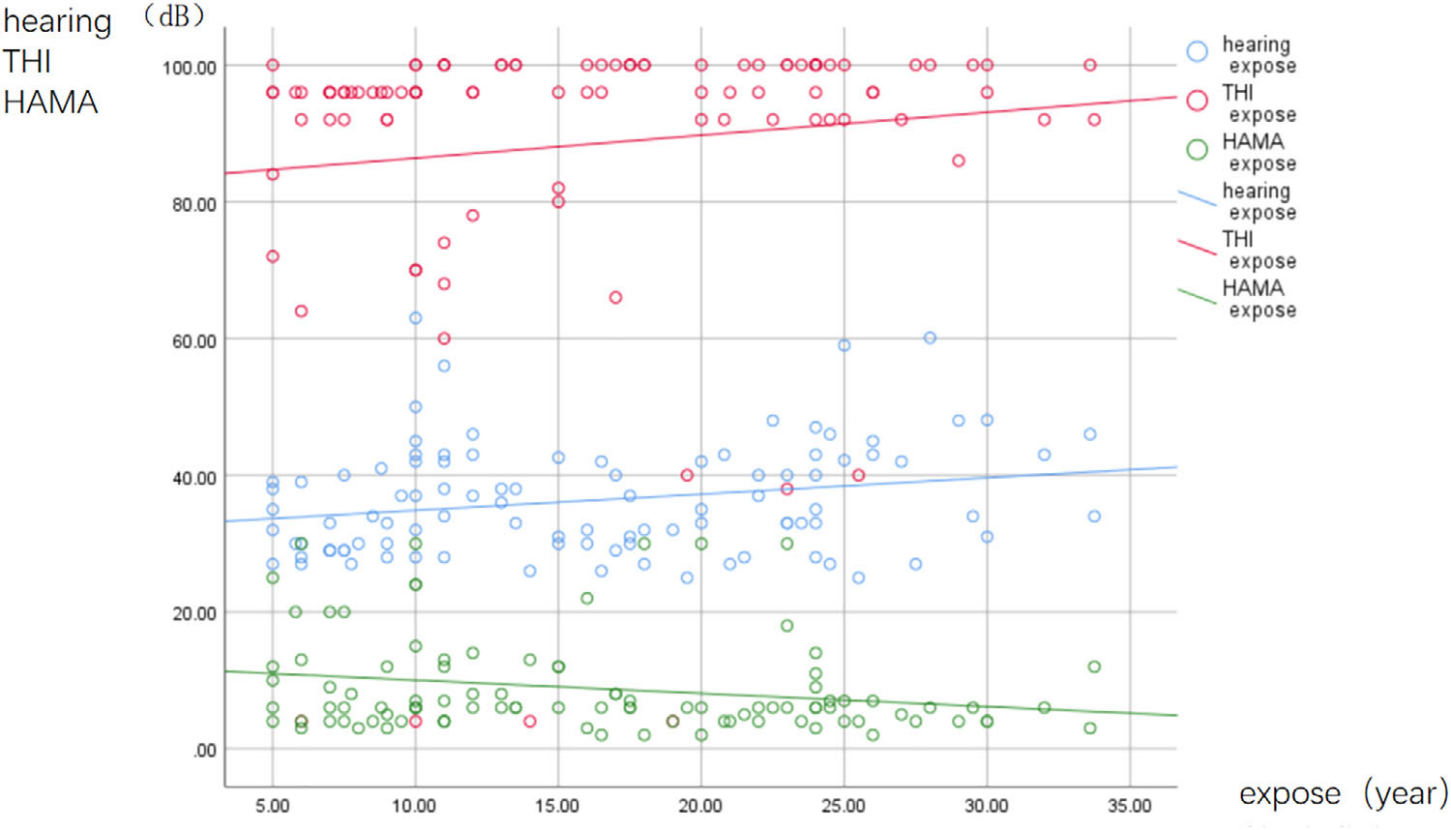
The correlation between noise exposure time and hearing threshold, tinnitus handicap inventory (THI), and Hamilton Anxiety Scale (HAMA) in noise‐induced hearing loss (NIHL) patients (hearing and exposure time: *r* = .233, *p* = .020; THI and exposure time: *r* = .122, *p* = .230; HAMA and exposure time: *r* = −.215, *p* = .033 [*p* < .05]).

**FIGURE 3:**
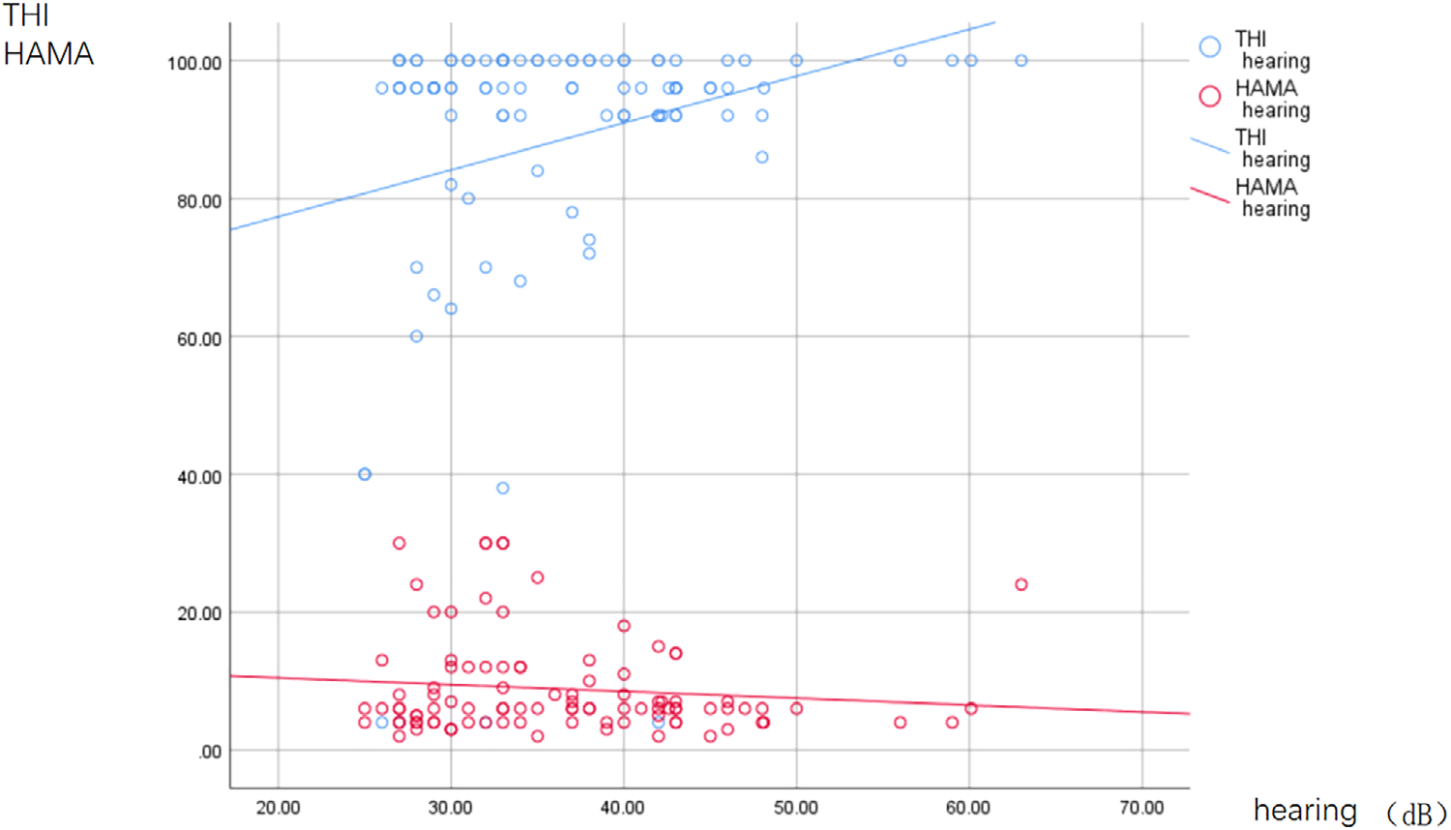
The correlation between hearing threshold and tinnitus handicap inventory (THI), Hamilton Anxiety Scale (HAMA) in noise‐induced hearing loss (NIHL) patients (THI and hearing: *r* = .253, *p* = .012; HAMA and hearing: *r* = −.112, *p* = .271 [*p* < .05]).

The study cohort comprised MP (*n* = 71; mean age: 45.52 ± 7.59 years; education level: 10.31 ± 2.01 years), RSP (*n* = 28; mean age: 47.32 ± 4.92 years; education level: 10.42 ± 2.12 years), and HCs (*n* = 75; mean age: 45.97 ± 7.82 years; education level: 11.36 ± 2.80 years) after a series of processing and elimination (11, 8, and 4 patients with undetermined, MP, and RSP, respectively, and 9 HCs) (Table 1). The ages, levels of education, or MMSE scores of the three groups were not different (*F* = 3.01, *p* > .05; *F* = .462, *p* > .05; *F* = .462, *p* > .05). The HAMA scores of the three groups were significantly different (*F* = 21.757, *p* < .05) (Table 1). The occupations of the MP and RSP were predominantly drilling and welding (noise environment above 85 dB). The THI scores of the MP and RSP groups were not different (*t* = −1.213, *p* > .05), but their hearing thresholds and noise exposure times differed (*t* = −11.416, *p* < .05; *t* = −2.690, *p* < .05) (Table 1). The Pearson correlation coefficient was.233 (*p* = .020) for noise exposure time and hearing threshold; −.215 (*p* = .033) for exposure time and HAMA scores; and.122 (*p* = .230) for noise exposure time and THI scores (Figure 2). The Pearson correlation coefficient was.253 (*p* = .012) for the hearing threshold and THI scores and −.112 (*p* = .271) for hearing loss and HAMA scores (Figure 3).

**TABLE 1:** Summary of characteristics of subjects (mean ± standard deviation) and correlation analysis.

	HC (*n* = 75)	MP (*n* = 71)	RSP (*n* = 28)	*F*/*t*	*p* Value
Age (year)	45.97 ± 7.82	45.52 ± 7.59	47.32 ± 4.92	.605	>.05
Education level (year)	11.21 ± 2.70	10.31 ± 2.01	10.42 ± 2.12	3.010	>.05
MMSE	28.85 ± 1.14	28.97 ± .90	29.07 ± 1.04	.516	>.05
Noise exposure time (year)	/	14.79 ± 7.37	19.58 ± 8.21	−2.690	.01^b^
Hearing threshold (superiority) (dB)	/	32.31 ± 4.43	46.5 ± 5.96	−11.416	<.001^b^
THI	/	92.42 ± 10.41	95.57 ± 4.10	−1.550	.180
HAMA	3.75 ± 1.08	9.58 ± 7.78	6.96 ± 4.56	21.757	<.001^a^

Abbreviations: HAMA, Hamilton Anxiety Scale; HC, health control; MMSE, Mini‐Mental State Examination; MP, mild patient; RSP, relatively severe patient; THI, tinnitus handicap inventory.

^a^ Comparison among three groups using one‐way analysis of variance, *p* < .05; post hoc correction analysis using Bonferroni, *p* < .0167 (HC vs. MP: *p* < .001; HC vs. RSP: *p* = .007; MP vs. RSP: *p* = .030).

^b^ Comparison between mild and relative severe groups using two independent sample *t* test, *p* < .05.

### 3.2 Whole‐brain voxel‐wise tract‐based statistical data and correlation analysis

**FIGURE 4:**
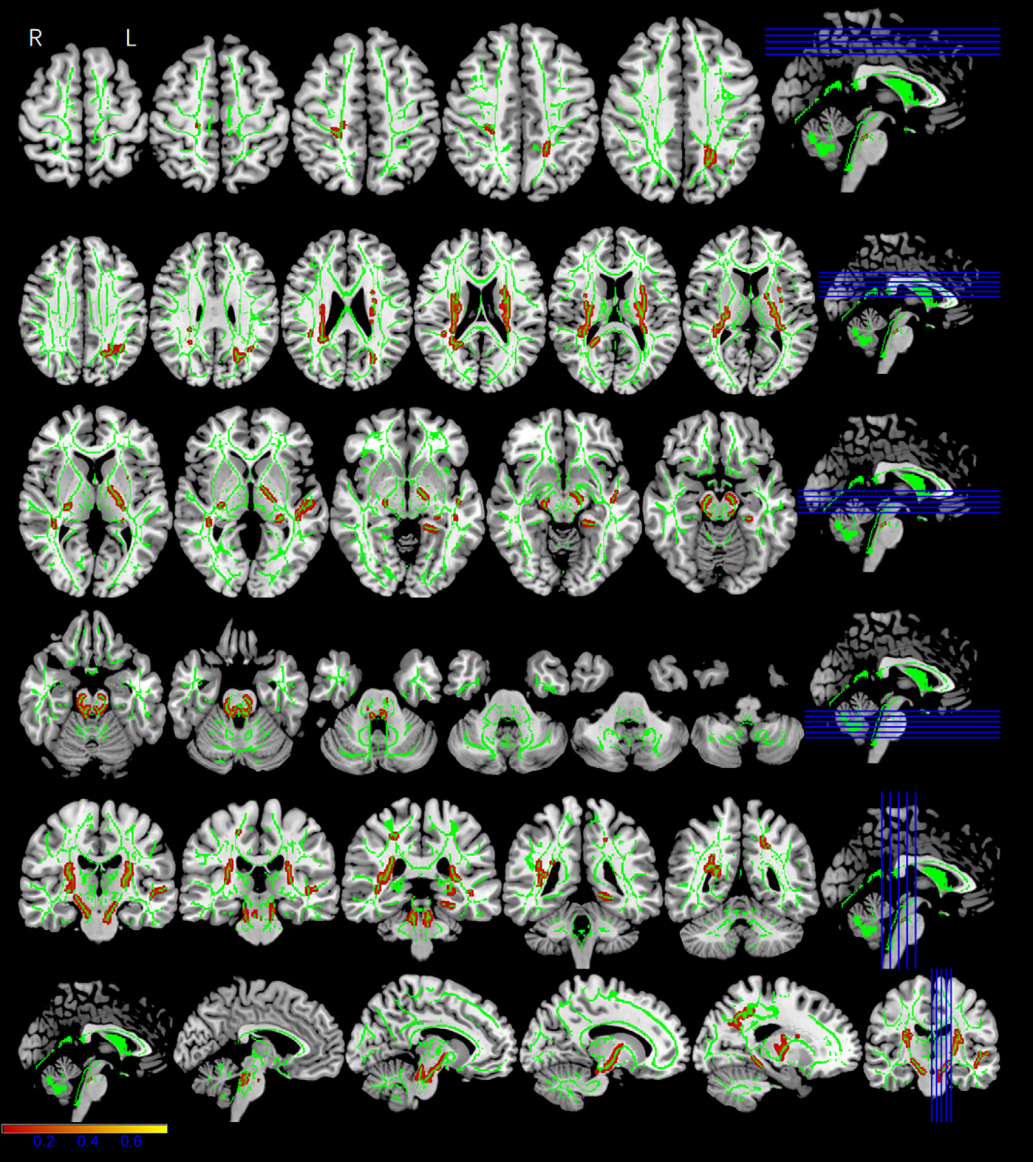
The fractional anisotropy (FA) maps showing significant differences among three groups. The red–yellow highlighted areas indicate lower FA values in the left inferior longitudinal fasciculus (ILF), left inferior fronto‐occipital fasciculus (IFOF), bilateral corticospinal tract (CST), right fronto‐pontine tract (FPT), right forceps major (FM), left superior longitudinal fasciculus (SLF) (temporal part), and left cingulum (hippocampus) (C‐H). The background image consists of the standard MNI T1‐weighted template at 1‐mm thickness and the FA skeleton (green); right‐hand column is corresponding section sagittal/coronal position.

**FIGURE 5:**
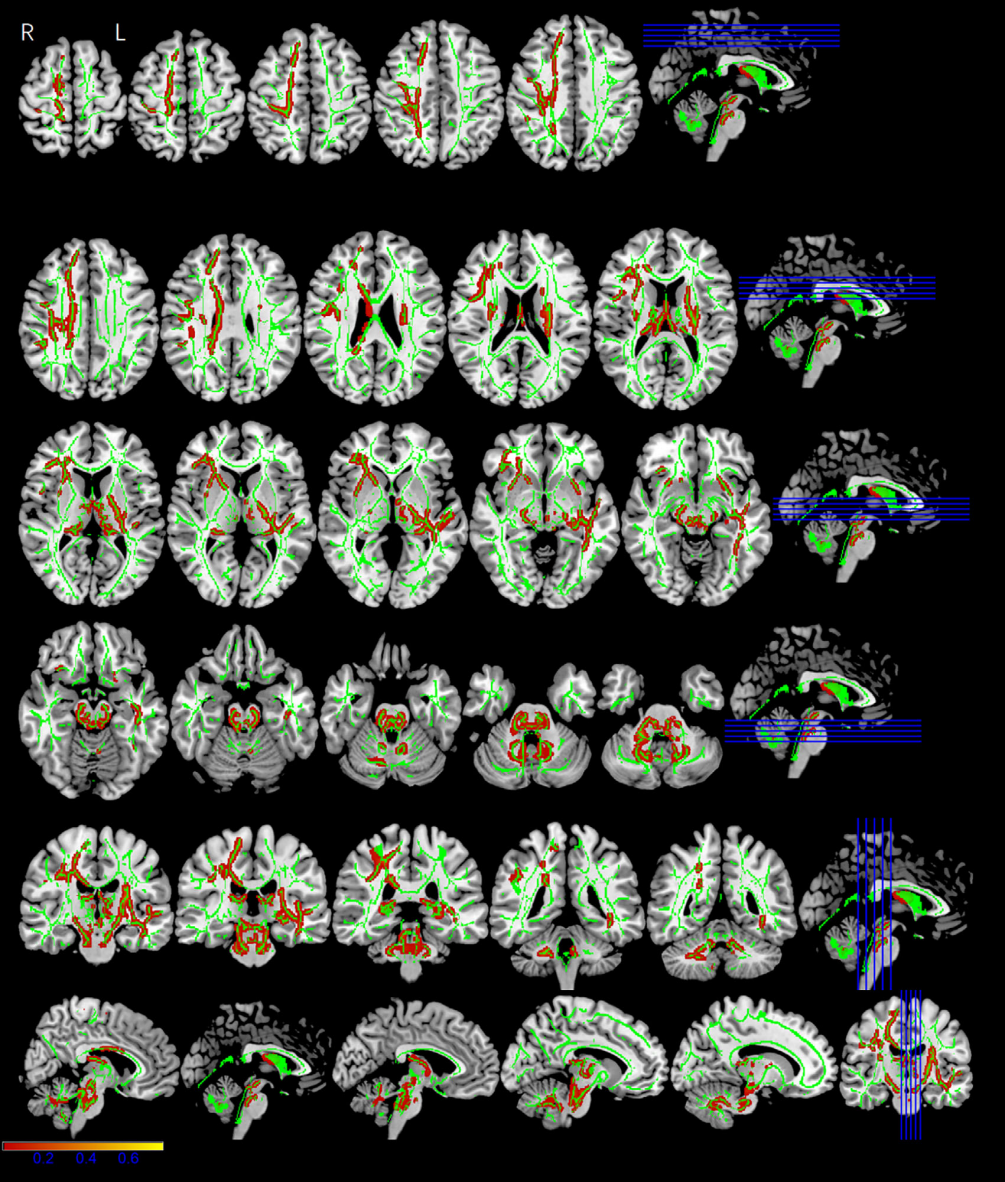
The axial diffusivity (AD) maps showing significant differences among three groups. The red–yellow highlighted areas indicate higher AD values in the left inferior longitudinal fasciculus (ILF), left inferior fronto‐occipital fasciculus (IFOF), right corticospinal tract (CST), right external capsule (EC), and right superior longitudinal fasciculus (SLF), they are significantly lower in left CST, right anterior thalamic radiation (ATR), and right superior cerebellar peduncle (SCP). The background image consists of the standard MNI T1‐weighted template at 1‐mm thickness and the fractional anisotropy (FA) skeleton (green) (the red–yellow scale represents regions of significant difference, with red representing regions of greatest difference); right‐hand column is corresponding section sagittal/coronal position.

**FIGURE 6:**
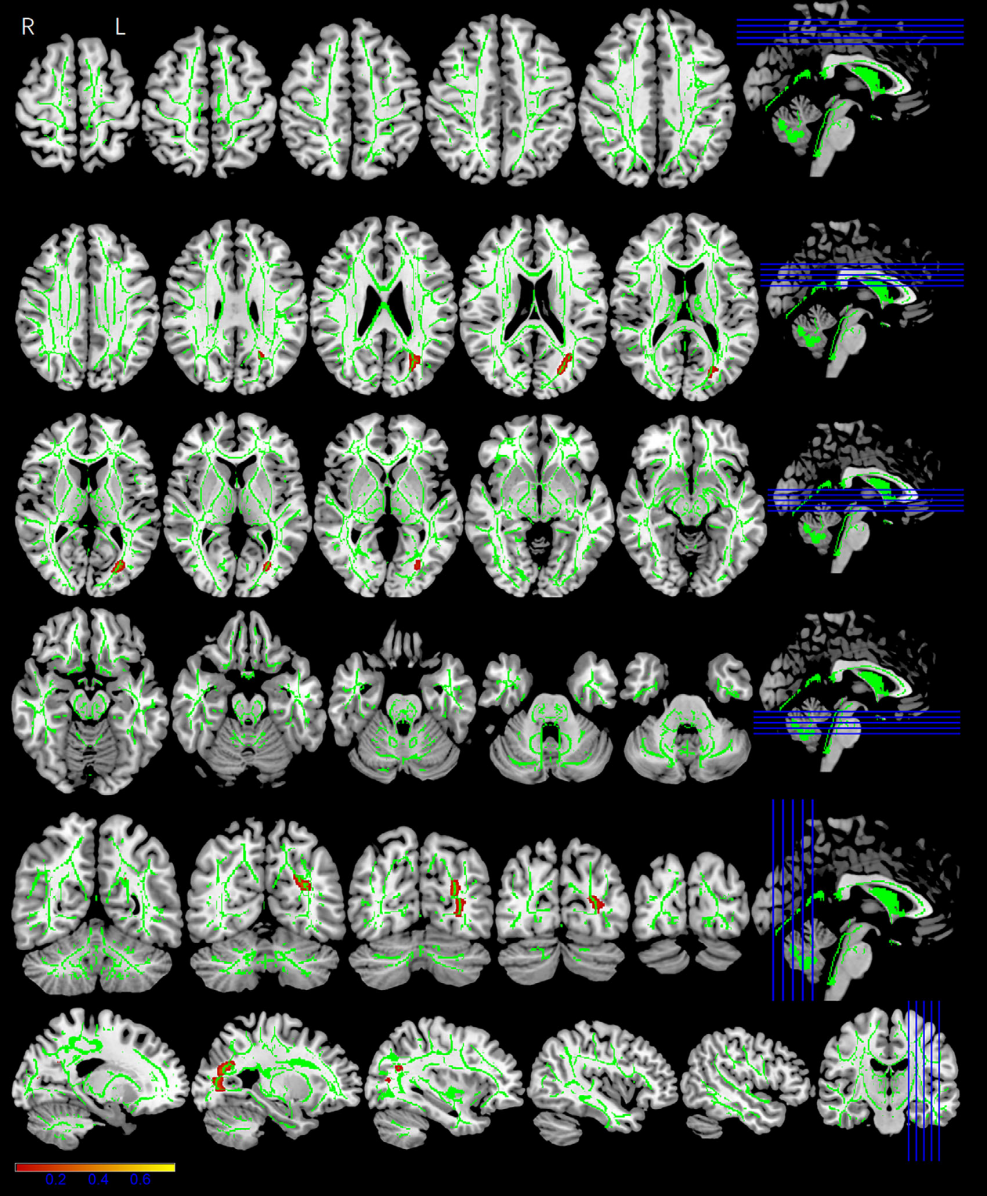
The mean diffusivity (MD) maps showing significant differences among three groups. The red–yellow highlighted areas indicate higher MD values in the left inferior longitudinal fasciculus (ILF) and left inferior fronto‐occipital fasciculus (IFOF). The background image consists of the standard MNI T1‐weighted template at 1‐mm thickness and the fractional anisotropy (FA) skeleton (green) (The red–yellow scale represents regions of significant difference, with red representing regions of greatest difference.); right‐hand column is corresponding section sagittal/coronal position.

**FIGURE 7:**
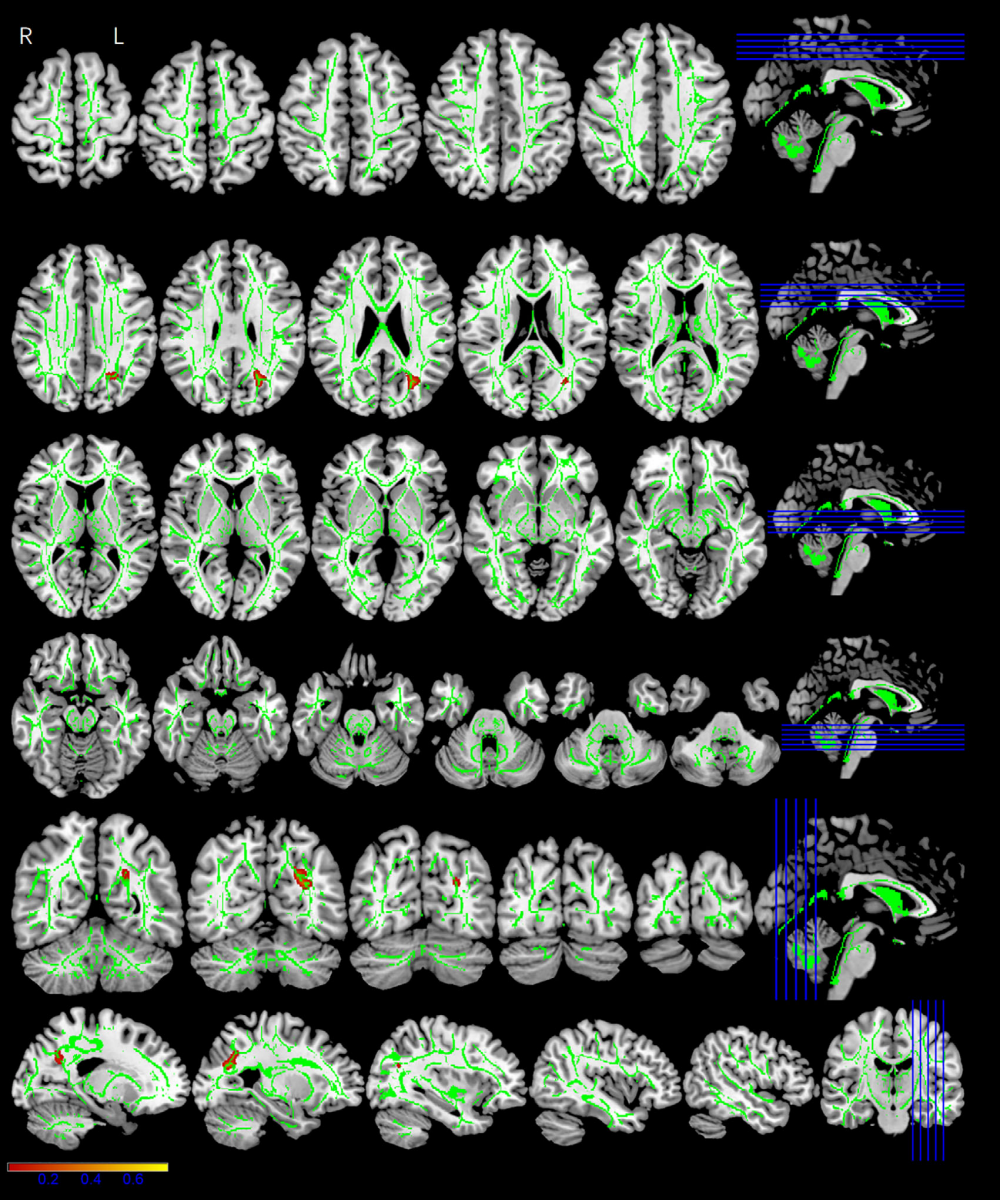
The radial diffusivity (RD) maps showing significantly differences among three groups. The red–yellow highlighted areas indicate higher RD values in the left inferior longitudinal fasciculus (ILF), left inferior fronto‐occipital fasciculus (IFOF). The background image consists of the standard MNI T1‐weighted template at 1‐mm thickness and the fractional anisotropy (FA) skeleton (green) (the red–yellow scale represents regions of significant difference, with red representing regions of greatest difference); right‐hand column was corresponding section sagittal/coronal position.

Whole‐brain tract‐based spatial statistics (TBSS) analysis showed significantly different FA, AD, MD, and RD maps for the three groups (*p* < .05, cluster voxels >100). All the DTI parameter values were statistically significant for the left inferior longitudinal fasciculus (ILF) and left inferior fronto‐occipital fasciculus (IFOF). The MP and RSP exhibited significantly lower FA (*F* = 20.981, *p* < .001) (Figure 4), higher AD (*F* = 9.119, *p* < .001) (Figure 5), higher MD (*F* = 16.646, *p* < .001) (Figure 6), and higher RD (*F* = 18.111, *p* < .001) (Figure 7), compared with the HCs (Table 2). The FA values were significantly lower in the bilateral corticospinal tract (CST) (L: *F* = 14.363/16.777, *p* < .001; R: *F* = 8.907, *p* < .001), right fronto‐pontine tract (FPT) (*F* = 22.510, *p* < .001), right forceps major (FM) (*F* = 10.252, *p* < .001), left superior longitudinal fasciculus (SLF) (temporal part) (*F* = 24.410, *p* < .001), and left cingulum (hippocampus) (C‐H) (*F* = 14.080, *p* < .001) of the MP and RSP than in those of the HCs (Figure 4, Table 2). The AD values demonstrated diverse changes. AD was significantly higher in the right CST (*F* = 6.984/5.995/5.761, *p* = .001/.003/.004), right external capsule (EC) (*F* = 14.921, *p* < .001), left IFOF (*F* = 9.119, *p* < .001), and right SLF (*F* = 10.23, *p* < .001) of the MP and RSP than in those of the HCs. However, it was significantly lower in the left CST (*F* = 5.580/7.853, *p* = .004/.001), right anterior thalamic radiation (ATR) (*F* = 12.919, *p* < .001), and right superior cerebellar peduncle (SCP) (*F* = 12.313, *p* < .001) of the MP and RSP than in those of the HCs (Figure 5, Table 2). Multiple comparisons of the groups showed that the FA, MD, and RD values of MP and RSP were significantly different from those of the HCs for all clusters (*p* < .05; Bonferroni correction: *p* < .0167), respectively. The FA, MD, and RD values of MP and RSP were not significantly different for any cluster (*p* > .0167). The AD values of the MP and HCs were significantly different for the right CST (*p* < .0167), right EC, right SCP, left IFOF, and right SLF. The AD values of the RSP and HCs were significantly different for the bilateral CST, right ATR, right EC, and right SLF (*p* < .0167). The AD values of the MP and RSP were significantly different for the right CST and right ATR (*p* < .0167) (Table 2).

**TABLE 2:** Summary of diffusion tensor imaging (DTI) parameters showing significant differences of subjects (control group, mild group, and the MS group).

		FMRIB58 coordinates				Three groups		M–HC	RSP–HC	MP–RSP
	Cluster voxels	*X*	*Y*	*Z*	Atlases (tract from JHU)	*F*	*p*	*p*	*p*	*p*
FA	1279	117	106	87	Corticospinal tract L	14.363	<.001	<.001^a^	<.001^a^	1.000
	1048	62	99	85	Corticospinal tract R	8.907	<.001	.002^a^	.002^a^	1.000
	604	116	63	99	IFOF L/ILF L	20.981	<.001	<.001^a^	<.001^a^	1.000
	370	85	94	48	Fronto‐pontine tract R	22.510	<.001	<.001^a^	<.001^a^	.984
	226	62	78	92	Forceps major R	10.252	<.001	<.001^a^	.013^a^	1.000
	176	146	108	73	Superior longitudinal fasciculus (temporal part) L	24.410	<.001	<.001^a^	<.001^a^	.858
	142	100	97	50	Corticospinal tract L	16.777	<.001	<.001^a^	.001^a^	1.000
	126	117	91	60	Cingulum (hippocampus) L	14.080	<.001	<.001^a^	.011^a^	1.000
AD	3293	118	115	90	Corticospinal tract L	5.580	.004	.099	.006^a^	.374
	2802	86	96	45	Corticospinal tract L	7.853	.001	.158	<.001^a^	.046
	2260	67	92	120	Corticospinal tract R	6.984	.001	.003^a^	1.000	.019
	1840	75	142	67	Anterior thalamic radiation R	12.919	<.001	.042	<.001^a^	.006^a^
	1647	72	109	108	Corticospinal tract R	5.995	.003	.056	.421	.004^a^
	184	120	137	66	External capsule R	14.921	<.001	<.001^a^	.001^a^	1.000
	154	77	116	60	Superior cerebellar peduncle R	12.313	<.001	<.001^a^	.019	1.000
	132	128	75	60	IFOF L/ILF L	9.119	<.001	<.001^a^	.192	.577
	104	46	84	106	Superior longitudinal fasciculus R	10.230	<.001	.016^a^	<.001^a^	.088
	103	61	115	89	Corticospinal tract R	5.761	.004	.058	.006^a^	.533
MD	146	120	63	94	IFOF L/ILF L	18.111	<.001	<.001^a^	<.001^a^	.295
RD	175	114	65	99	IFOF L/ILF L	16.646	<.001	<.001^a^	<.001^a^	1.000

*Note*: Comparison among three groups using *t* one‐way analysis of variance, *p* < .05; voxels >100.

Abbreviations: AD, axial diffusivity; FA, fractional anisotropy; IFOF, inferior fronto‐occipital fasciculus; ILF, inferior longitudinal fasciculus; JHU, Johns Hopkins University; L, left; MP–HC, the comparison between the mild patient group and the health control group; MD, mean diffusivity; MP–RSP, the comparison between the mild patient group and the relatively severe patient group; R, right; RD, radial diffusivity; RSP–HC, the comparison between the relatively severe patient group and the health control group.

^a^ Bonferroni test was used for multiple comparisons among groups, *p* < .05; voxels >100 (Bonferroni correction: *p* < .0167).

The FA and HAMA scores of the three groups were negatively correlated in the left IFOF and ILF, right FPT, left SLF, and left C‐H (*r* = .168, *p* = .027; *r* = .182, *p* = .016; *r* = .215, *p* = .004; *r* = .248, *p* = .001, respectively) (Table 3). There were significant positive correlations between AD and the HAMA scores for the left IFOF/ILF and right EC (*r* = .170, *p* = .025; *r* = .150, *p* = .049) (Table 4). The RD, MD, and HAMA scores for the brain regions of the three groups were significantly positively correlated with the IFOF/ILF on the left side (*r* = .199, *p* = .009; *r* = .161, *p* = .034) (Table 3).

**TABLE 3:** Summary of diffusion tensor imaging (DTI) parameters showing significant differences of subjects (mean ± standard deviation) and correlation analysis.

	Cluster voxels	FMRIB58 coordinates			Atlases (tract from JHU)	HC (*n* = 75)	MP (*n* = 71)	RSP (*n* = 28)	Hearing		Expose noise time		HAMA	
		*X*	*Y*	*Z*					*r*	*p*	*r*	*p*	*r*	*p*
FA	1279	117	106	87	Corticospinal tract L	.684 ± .022	.658 ± .030	.660 ± .030	−.059	.562	.066	.513	−.083	.277
	1048	62	99	85	Corticospinal tract R	.608 ± .022	.593 ± .030	.587 ± .021	−.054	.595	.157	.120	.004	.958
	604	116	63	99	IFOF L/ILF L	.487 ± .025	.457 ± .026	.456 ± .030	−.024	.813	−.003	.980	−.168	.027^a^
	370	85	94	48	Fronto‐pontine tract R	.720 ± .016	.695 ± .027	.700 ± .022	.076	.458	.033	.743	−.182	.016^a^
	226	62	78	92	Forceps major R	.674 ± .025	.651 ± .033	.653 ± .035	.078	.444	.080	.431	−.146	.055
	176	146	108	73	Superior longitudinal fasciculus (temporal part) L	.399 ± .029	.368 ± .028	.360 ± .026	−.055	.590	−.060	.556	−.215	.004^a^
	142	100	97	50	Corticospinal tract L	.652 ± .019	.632 ± .026	.631 ± .020	−.056	.584	.034	.739	−.118	.121
	126	117	91	60	Cingulum (hippocampus) L	.480 ± .027	.439 ± .053	.449 ± .040	.025	.804	−.042	.680	−.248	.001^a^
AD	3293	118	115	90	Corticospinal tract L	.00140 ± .000116	.00142 ± .000130	.00137 ± .000129	−.197	.051	.060	.552	.033	.669
	2802	86	96	45	Corticospinal tract L	.00104 ± .000100	.00102 ± .000100	.00103 ± .000122	.037	.716	.068	.5032	.029	.702
	2260	67	92	120	Corticospinal tract R	.00141 ± .000466	.00140 ± .000412	.00127 ± .000298	−.006	.949	.043	.669	−.005	.943
	1840	75	142	67	Anterior thalamic radiation R	.00094 ± .000110	.00095 ± .000104	.00098 ± .000106	.117	.248	−.093	.358	−.016	.835
	1647	72	109	108	Corticospinal tract R	.00097 ± .000088	.00095 ± .000102	.00093 ± .000103	.081	.425	.046	.649	.036	.635
	184	120	137	66	External capsule R	.00132 ± .000139	.00128 ± .000097	.00126 ± .000142	−.008	.939	−.005	.957	.150	.049^a^
	154	77	116	60	Superior cerebellar peduncle R	.00089 ± .000097	.00100 ± .000389	.00093 ± .000197	−.047	.646	−.205	.042^a^	−.119	.119
	132	128	75	60	IFOF L/ILF L	.00098 ± .000083	.00100 ± .000077	.00100 ± .000077	−.043	.674	.052	.611	.170	.025^a^
	104	46	84	106	Superior longitudinal fasciculus R	.00166 ± .000163	.00161 ± .000159	.00162 ± .000239	−.118	.092	.157	.120	.072	.347
	103	61	115	89	Corticospinal tract R	.00133 ± .00008	.00136 ± .00010	.00135 ± .00009	−.007	.945	−.061	.551	.071	.355
MD	146	120	63	94	IFOF L/ILF L	.00080 ± .000033	.00083 ± .000036	.00084 ± .000031	.041	.688	−.019	.856	.199	.009^a^
RD	175	114	65	99	IFOF L/ILF L	.00057 ± .000044	.00061 ± .000040	.00061 ± .000039	−.001	.989	−0020	.848	.161	.034^a^

Abbreviations: AD, axial diffusivity; FA, fractional anisotropy; HAMA, Hamilton Anxiety Scale; IFOF, inferior fronto‐occipital fasciculus; ILF, inferior longitudinal fasciculus; JHU, Johns Hopkins University; L, left; MD, mean diffusivity; MP–HC, the comparison between the mild patient group and the health control group; MP–RSP: the comparison between the mild patient group and the relatively severe patient group; R, right; RD, radial diffusivity; RSP–HC: the comparison between the relatively severe patient group and the health control group.

^a^ Correlation analysis by Spearson correlation analysis, *p* < .05.

**TABLE 4:** Summary of region of interest–diffusion tensor imaging (ROI–DTI) parameters showing significant differences of subjects (mean ± standard deviation) and correlation analysis.

	ROI	HC (*n* = 75)	MP (*n* = 71)	RSP (*n* = 28)	Three groups		M‐HC^a^	RS‐HC^a^	M‐RS^a^	Hearing		Expose noise time		HAMA	
					*F*	*p*	*p*	*p*	*p*	*r*	*p*	*r*	*p*	*r*	*p*
FA	LHG	.134 ± .011	.134 ± .010	.135 ± .010	.166	.847	>.05	>.05	>.05	.036	.724	.147	.147	−.017	.828
	RHG	.133 ± .014	.133 ± .013	.131 ± .012	.323	.725	>.05	>.05	>.05	−.086	.400	−.055	.587	.165	.030^b^
AD	LHG	.00134 ± .00018	.00135 ± .00013	.00137 ± .00017	.541	.583	>.05	>.05	>.05	.229	.023^b^	.125	.219	.081	.285
	RHG	.00128 ± .00016	.00131 ± .00015	.00132 ± .00015	.573	.565	>.05	>.05	>.05	.156	.124	.099	.331	.073	.337
MD	LHG	.00118 ± .00015	.00119 ± .00012	.00121 ± .00013	.439	.645	>.05	>.05	>.05	.231	.022^b^	.113	.267	.080	.292
	RHG	.00115 ± .00016	.00117 ± .00014	.00118 ± .00013	.435	.648	>.05	>.05	>.05	.159	.116	.102	.315	.050	.510
RD	LHG	.00113 ± .00016	.00113 ± .00011	.00116 ± .00015	.383	.682	>.05	>.05	>.05	.230	.022^b^	.105	.302	.080	.296
	RHG	.00109 ± .00015	.00110 ± .00013	.00111 ± .00013	.365	.695	>.05	>.05	>.05	.160	.114	.103	.309	.038	.623

*Note*: Comparison among three groups using *t* one‐way analysis of variance, *p* < .05; voxels >100.

Abbreviations: AD, axial diffusivity; FA, fractional anisotropy; HAMA, Hamilton Anxiety Scale; HG, Heschl's gyrus; L, left; MD, mean diffusivity; MP–HC, the comparison between the mild patient group and the health control group; MP–RSP, the comparison between the mild patient group and the relatively severe patient group; R, right; RD, radial diffusivity; RSP–HC, the comparison between the relatively severe patient group and the health control group.

^a^ Bonferroni test was used for multiple comparisons between groups, *p* < .05; voxels >100 (Bonferroni correction: *p* < .0167).

^b^ Correlation analysis by the Spearson correlation analysis, *p* < .05.

### 3.3 Region of interest and correlation analyses

There were no statistically significant differences among the bilateral auditory cortex ROIs of the three groups. Multiple comparisons between the groups showed no statistical significance (Table 4). The FA of the right auditory cortex was positively correlated with the HAMA score (*r* = .165, *p* = .030), and the AD, MD, and RD of the left auditory cortex were negatively correlated with the hearing threshold (*r* = .229, *p* = .023; *r* = .231, *p* = .022; *r* = .230, *p* = .022, respectively) (Table 4).

## 4 DISCUSSION

The main occupation of the participants of the study was mining. Their noise exposure intensity exceeded 85 dB, which is higher than the national occupational standard, ignoring other recreational noises in any group (e.g., personal music players) and other sources of noise that were not produced by drilling or welding in this study. The hearing threshold was positively correlated with noise exposure time. This confirmed that long‐term noise exposure can cause hearing damage, which is consistent with the damage mechanism of NIHL (Fettiplace, 2017). The HAMA score was negatively correlated with noise exposure time, which suggests that patients with NIHL during long‐term exposure to the noise environment adapt to the noise, tinnitus, and hearing loss, and their anxiety gradually decreases. This was different from the patterns of anxiety, fear, manic disorder, frustration in life, and maladjustment in patients with acute sudden deafness (Wang et al., 2018). After a long period of adjustment and adaptation, patients gradually accept the state of deafness. The reduction of anxiety may be caused by the increase in the psychological identity and psychological adaptation of the patient to deafness, leading to “co‐survival” with deafness (Qi et al., 2015). However, the HAMA scores of the patients with MP and RSP were higher than those of the HCs, indicating that noise and hearing loss can affect mental health even though patients with NIHL have environmental adaptation. This is consistent with the report of Pace and Zhang (2013). Therefore, preventive measures are important to preserve the good mental health of patients with NIHL.

### 4.1 Whole‐brain tract‐based spatial statistics

The DTI parameters included the FA, MD, AD, and RD. FA represents the degree of tissue anisotropy, which is related to the integrity of WM fibers (Steele et al., 2013), and reflects fiber density, axon diameter, and the degree of WM myelination (Poletti et al., 2015). MD reflects fiber demyelination, decreased cell density, or increased extracellular volume (Tang et al., 2007). Therefore, the MD quantifies the overall diffusion of water molecules within a region. AD and RD represent axon injury and myelin loss, respectively. Partial anisotropy combines the two and represents the directivity or genus of fibers in the microstructure (Basser & Pierpaoli, 1996). According to Wang et al. (2022), a machine learning model combining the DTI parameters (FA, AD, MD, and RD) into a single feature performed better than that using a single‐DTI parameter to evaluate the WM fiber bundles. Therefore, this model can be used to evaluate the microstructure of WM fiber bundles. In this study, the main fiber bundles of the brain regions included the ILF and IFOF. FA was significantly negatively correlated with HAMA scores, whereas AD, RD, and MD were significantly positively correlated with the HAMA scores of the three groups.

The ILF has been suggested to play major roles in several brain functions associated with selected structures (Herbet et al., 2018), including the anterior temporal and occipital lobes. Injury to the ILF pathway leads to defects in object recognition, which is involved in visual processing, visual processing tasks, language, semantic function, emotional regulation, reading, and neuropsychiatric states (Boets et al., 2018). The IFOF is one of the longest associative fibers in the human brain; it connects the occipital and frontal lobes and plays key roles in the functions of several brain structures, such as the auditory, visual, and prefrontal cortices, which are critical to executive function (Ivanova et al., 2016; Martino et al., 2010). Therefore, IFOF integrity is required for reading, visual processing, and language comprehension. All DTI parameter values (FA, MD, AD, and RD) for the left ILF and left IFOF of the MP and RSP were significantly different from those of the HCs, which may be related to the left dominant hemisphere lateralization of the audition and language center (Huang et al., 2020). Leng et al. (2016) also reported a similar result; the changes of the FA and MD values tended to be left‐sided in the occipital lobe segment of the IFOF. Partially consistent with Wang et al. (2019), lower FA values were found for the ILF and IFOF in SNHL children. Qi et al. (2019) observed decreased FA and AD and increased RD in several brain areas (ILF and IFOF) in middle‐aged patients who were first recruited and diagnosed with SNHL. Similarly, Luan et al. (2019) found that patients with long‐term bilateral SNHL had lower FA values in the IFOF, and they were positively correlated with the mean hearing thresholds. Therefore, combined with the characteristics of various DTI parameters, patients with NIHL may suffer from the dysfunction of brain structural connections caused by auditory deprivation under long‐term unprotected exposure in a noisy environment. This may include less myelination, axonal loss, and/or fewer fiber projections.

The whole‐brain tract‐based spatial statistical analysis in this study showed lower FA in the bilateral CST, right FPT, right FM, left SLF (temporal part), and left C‐H of the MP and RSP than in those of the HCs. These brain structures were the association and projection fibers between the GM structures (Wang et al., 2019). Regardless of whether early hearing deprivation in children or persistently deaf individuals can affect motor fibers (Jiang et al., 2019; Wang et al., 2019), damage to the CST may affect the related motor network. Some studies (Dresang et al., 2021) believe that FPT damage affects frontal lobe function and causes emotional disorders, which is consistent with the significant correlation between the change in the FPT FA value and HAMA score in this study. Li et al. (2020) speculated that the anxiety of patients was related to damage to the FM fiber bundle. A study on senile deafness with cognitive impairment (Rigters et al., 2018) found a lower FA in the SLF. Our study excluded senile deafness and cognitive impairment, but the patients with NIHL still showed cognitive‐related fiber bundle changes. The C‐H is the collateralization of the limbic system and is related to emotional regulation and cognitive function (Ren et al., 2022). Therefore, changes in FA in these brain regions suggest that patients with NIHL exposed to noise for a long time without protection or ineffective protection may experience hearing impairment, hearing deprivation, loss of peripheral auditory input, or abandonment of the relevant auditory cortex, ultimately leading to dysfunctional connectivity among brain structures, which further affects emotional disorders and cognitive function.

The AD findings may further support this hypothesis; it was significantly higher in the right CST, right EC, left IFOF, and right SLF and lower in the left CST, right ATR, and right SCP of the MP and RSP than in those of the HCs. This is consistent with prior reports (Husain et al., 2011; Profant et al., 2014) that decreased AD may indicate axon damage. Jiang et al. (2019) found that higher AD may be attributed to abnormal axonal maturity, such as lower axonal density and caliber. Fine et al. (2005) summarized the two main human brain abilities of “cross‐modal plasticity” and “compensatory hypertrophy” (the main parts of the visual cortex undergo proliferation and remodeling). Some studies on aging (Bender et al., 2016; Cox et al., 2016) have also suggested diverse changes in AD, and postmortem histology has confirmed that this change in the pattern of WM fiber bundles is related to the degeneration and deformation of axons and myelin. These diverse changes in AD indicate that axonal damage, proliferation, and remodeling occur in patients with NIHL. This conforms to the functional plasticity setting of the human brain and highlights the involvement of these brain regions in cognitive and emotional regulation functions.

Subsequent multiple comparisons of the groups showed statistically significant differences in the DTI parameters of the patients with NIHL with different grades and HCs. There were no significant differences in the FA, MD, or RD values of the MP and RSP for all the brain regions of the fiber bundle, but there were statistical differences in the AD values of the right ATR and CST. These results are slightly different from those of our previous amplitude of low‐frequency fluctuation (ALFF) study (Huang et al., 2020). Therefore, the hearing impairment induced by noise exposure was not highly correlated with the severity of the disease. The correlation between the level of hearing loss and the time of noise exposure also confirmed this; damage to the WM microstructure had already occurred during the early stages of NIHL. Therefore, the effective prevention of NIHL is important, and the degree of disease development will continue to impair NIHL‐related secondary diseases, such as WM fiber bundles involving mood, sleep, and depression.

### 4.2 Region of interest analysis

Several studies (Husain et al., 2011; Park et al., 2018) have inferred that the auditory cortex and WM tract integrity are related to hearing loss reduction. However, our ROI “Auditory Cortex” did not demonstrate this effect in HG. There were no statistically significant differences between the bilateral auditory cortex ROIs of the three groups. Aldhafeeri et al. (2012) and Gunbey et al. (2017) observed similar effects on the auditory cortical WM. This result may be explained by the spatially coarse feature of diffusion imaging, which affects its ability to detect NIHL‐related changes in this area. These results suggest that the spatial cortex may not be easily detected, although NIHL‐related changes in other WM tracts may be present or auditory cortex dysfunction may occur later. The previous whole‐brain TBSS analysis of DTI parameters in different brain regions did not significantly correlate with the hearing threshold, but correlation analysis of the ROIs showed that AD, MD, and RD in the left auditory cortex were positively correlated with the hearing threshold. The increase in the AD, MD, and RD values implied the possibility of compensatory remodeling of the auditory cortex and left dominance. Therefore, changes in the WM fiber bundle and axonal myelin sheath of the auditory cortex are related to the degree of hearing loss. Similarly, the FA value of the right auditory cortex showed a significant positive correlation with the HAMA scores during the ROI correlation analysis. This state of anxiety may compensate for damage to the integrity of the WM fiber bundles. The inconsistencies in these results may be due to the heterogeneity of cortical auditory damage and subcortical matter WM fiber tract damage caused by exposure to noise and hearing loss, which is speculated to be a complex mechanism that needs to be explored in further studies. Some reasons for these effects are the potentially large heterogeneity of the noise exposure times and hearing loss levels of patients with auditory damage, which also impacts the integrity of the auditory cortex (Schwarz et al., 2014).

### 4.3 Limitations

This study has a few limitations. First, the study included a small sample of RSP. Therefore, the results should be interpreted with caution. Future studies with larger samples may clarify the functional abnormalities involved in dysfunction related to NIHL. Second, we did not assess other recreational noises for any of the groups (such as personal music players) or other sources of noise that were not drilling or welding. Third, the neuropsychological tests were rare, and future research should include specific cognitive or emotional function tests related to NIHL. Fourth, the incidence of tinnitus should be evaluated. Tinnitus is a known accompanying symptom, and we found it difficult to assess the variation, time since onset, and severity in our study. The mechanisms underlying these findings require further investigations.

## 5 CONCLUSION

In this study, all patients with acquired NIHL had a long history of hearing loss during prolonged noise exposure. We excluded other hearing and cognitive impairments to make our results more reliable and internally consistent. In summary, DTI may be used as a noninvasive tool to investigate brain microstructural connectivity in vivo. This study found abnormal integrity of the WM fibers in multiple brain regions, especially the left ILF and left IFOF. This suggests that WM microstructural alterations (myelin and axonal dysfunction) in patients with NIHL may be due to specific structural changes in the brain or functional neuroplasticity with the reorganization of the noisy environment.

## AUTHOR CONTRIBUTIONS

**Huang Ranran**: Conceptualization; data curation; formal analysis; writing—review and editing; writing—original draft. **Wang Aijie**: Conceptualization; writing—review and editing; methodology; software. **Zhang Yafei**: Conceptualization; visualization; investigation. **Li Guochao**: Conceptualization. **Lin Yi**: Conceptualization. **Ba Xinru**: Conceptualization. **Li Yunxin**: Conceptualization. **Bao Xianghua**: Conceptualization; supervision. **Zhang Guowei**: Conceptualization; supervision; validation; writing—review and editing; project administration; resources; funding acquisition.

## CONFLICT OF INTEREST STATEMENT

The authors declare no conflicts of interest.

## FUNDING INFORMATION

There was no funding for this study.

### PEER REVIEW

The peer review history for this article is available at https://publons.com/publon/10.1002/brb3.3479.

## Data Availability

The data that support the findings of this study are available on request from the corresponding author. The data are not publicly available due to privacy or ethical restrictions.
